# Supplementary material for: Incidence and influencing factors related to social isolation among HIV/AIDS patients: Protocol for a systematic review and meta-analysis
Source: PLoS One. 2024 Jul 25;19(7):e0307656. doi: 10.1371/journal.pone.0307656 (PMC11271913; doi:10.1371/journal.pone.0307656)
Supplement: S2 File — (DOCX) [file pone.0307656.s002.docx]

**S2 File. Search strategy of Pubmed database**

| **Components** | **Search items** |
| --- | --- |
| #1 | ((((((((((((HIV[MeSH Terms]) OR (Acquired Immunodeficiency Syndrome[MeSH Terms])) OR (Human Immunodeficiency Virus[Title/Abstract])) OR (Human T Cell Lymphotropic Virus Type III[Title/Abstract])) OR (Human T-Cell Lymphotropic Virus Type III[Title/Abstract])) OR (LAV-HTLV-III[Title/Abstract])) OR (Lymphadenopathy-Associated Virus[Title/Abstract])) OR (Lymphadenopathy Associated Virus[Title/Abstract])) OR (Human T Lymphotropic Virus Type III[Title/Abstract])) OR (AIDS[Title/Abstract])) OR (Acquired Immune Deficiency Syndrome Virus[Title/Abstract])) OR (HTLV-III[Title/Abstract])) OR (Acquired Immuno Deficiency Syndrome[Title/Abstract]) |
| #2 | (((((((((((((((((Social Isolation[MeSH Terms]) OR (Social Isolation[Title/Abstract])) OR (Social Separation[Title/Abstract])) OR (Social segregation[Title/Abstract])) OR (Social quarantine[Title/Abstract])) OR (Social insulation[Title/Abstract])) OR (Social seclusion[Title/Abstract])) OR (Social shielding[Title/Abstract])) OR (Social partitioning[Title/Abstract])) OR (Social solitariness[Title/Abstract])) OR (Social aloneness[Title/Abstract])) OR (Social remoteness[Title/Abstract])) OR (Social loneliness[Title/Abstract])) OR (Social friendlessness[Title/Abstract])) OR (isolation stress[Title/Abstract])) OR (Solitude[Title/Abstract])) OR (social deprivation[Title/Abstract])) OR (psychosocial deprivation[Title/Abstract]) |
| #3 | (((((((((((Epidemiology[MeSH Terms]) OR (epidemiology[Subheading])) OR (incidence[MeSH Terms])) OR (Prevalence[MeSH Terms])) OR (Epidemi*)) OR (Incidence*)) OR (Frequency)) OR (Surveillance)) OR (Occurrence)) OR (outbreaks)) OR (Prevalence)) OR (Endemics) |
| #4 | ((((((((((Risk Factors[MeSH Terms]) OR (Risk Factor*[Title/Abstract])) OR (Factor, Risk[Title/Abstract])) OR (Factors, Risk[Title/Abstract])) OR (Population at Risk[Title/Abstract])) OR (Risk, Population at[Title/Abstract])) OR (Populations at Risk[Title/Abstract])) OR (Risk, Populations at[Title/Abstract])) OR (influenc*[Title/Abstract])) OR (Affecting Factor*[Title/Abstract])) OR (Factor*[Title/Abstract]) |
| #5 | #3 OR #4 |
| #6 | #1 AND #2 AND #5 |
